# On the assessment of coordination between upper extremities: towards a common language between rehabilitation engineers, clinicians and neuroscientists

**DOI:** 10.1186/s12984-016-0186-x

**Published:** 2016-09-08

**Authors:** Camila Shirota, Jelka Jansa, Javier Diaz, Sivakumar Balasubramanian, Stefano Mazzoleni, N. Alberto Borghese, Alejandro Melendez-Calderon

**Affiliations:** 1 Department of Health Sciences and Technology, ETH Zurich, Zurich, 8092 Switzerland; 2 University Medical Centre Ljubljana, Division of Neurology, Zaloska 2, 1000 Ljubljana, Slovenia; 3 CEIT-IK4, Paseo Mikelegeti, 48, 20009 San Sebastián, Spain; 4 Department of Bioengineering, Christian Medical College, Bagayam, Vellore, 632002 India; 5 The BioRobotics Institute, Scuola Superiore Sant’Anna, 56025 Pontedera, Italy; 6 Applied Intelligent Systems Laboratory (AIS-Lab), Department of Computer Science, University of Milano, Via Celoria 20, Milan, Italy; 7 Department of Physical Medicine and Rehabilitation, Northwestern University, Chicago, IL 60611 USA; 8 Hocoma AG, Volketswil, 8604 Switzerland

**Keywords:** Interlimb coordination, Upper limbs, Assessment, Robot-aided, Sensor-based

## Abstract

Well-developed coordination of the upper extremities is critical for function in everyday life. Interlimb coordination is an intuitive, yet subjective concept that refers to spatio-temporal relationships between kinematic, kinetic and physiological variables of two or more limbs executing a motor task with a common goal. While both the clinical and neuroscience communities agree on the relevance of assessing and quantifying interlimb coordination, rehabilitation engineers struggle to translate the knowledge and needs of clinicians and neuroscientists into technological devices for the impaired. The use of ambiguous definitions in the scientific literature, and lack of common agreement on what should be measured, present large barriers to advancements in this area. Here, we present the different definitions and approaches to assess and quantify interlimb coordination in the clinic, in motor control studies, and by state-of-the-art robotic devices. We then propose a taxonomy of interlimb activities and give recommendations for future neuroscience-based robotic- and sensor-based assessments of upper limb function that are applicable to the everyday clinical practice. We believe this is the first step towards our long-term goal of unifying different fields and help the generation of more consistent and effective tools for neurorehabilitation.

## Background

This work was developed as part of the project “State of the Art Robot-Supported assessments (STARS)” in the frame of the COST Action TD1006 “European Network on Robotics for NeuroRehabilitation” [1]. The goal of STARS is to give neurorehabilitation clinical practitioners and scientists recommendations for the development, implementation, and administration of different indices of robotic assessments, grounded on scientific evidence.

Well-coordinated movements are a characteristic feature of well-developed motor behavior. From neuroscientists to clinicians, quantifying coordination of an individual is of critical importance. Not only does this help in understanding the neurophysiological components of movement (neuroscience field), but it can also help us identify and assess underlying neurological problems of a patient with movement disorders, and guide therapeutic interventions (clinical field).

The term ‘coordination’ is so strongly ingrained in our common language that we do not typically stop to think about the key underlying features that characterize good and bad coordination–even though we can all distinguish the well-coordinated movements of a trained dancer from those of a novice. What exactly is meant by coordination? And how should it be measured? Addressing these questions is particularly difficult when considering such an abstract concept, which encompasses many different aspects that are not straightforward to define formally.

Indeed, coordinated movements are multidimensional and require the organization of multiple subsystems, e.g., eye-hand coordination [2], intersegmental coordination [3], intralimb coordination [4], interlimb coordination [5]. Given the multiple connotations and associations to the word *coordination*, in this paper, we attempt to summarize how coordination between upper extremities-a form of interlimb coordination-is interpreted and measured by clinicians, neuroscientists and rehabilitation engineers.

As the reader will see in the following pages, the descriptors of *interlimb coordination* and how it is assessed vary considerably from field to field, and even within a field. This lack of a common language and standard terminology is a huge barrier to relate the observations from different fields, hindering the understanding and discussion needed to move forward. Further, such definitions are critical for engineers working in translational neurorehabilitation, who harness knowledge from basic and clinical neuroscience to produce technological tools (e.g., robotic devices, instrumented tools) to aid clinicians in their everyday practice. The lack of a common understanding has fostered the use of dozens of ad-hoc algorithms and assessment tools (see section 3), most of which have had limited transfer to everyday clinical applications.

Our long-term goal is to standardize the administration of robotic-and sensor-based assessments of sensory-motor function. Towards this end, we present a summary of different ways in which interlimb coordination has been studied and quantified. We start by presenting a general overview of why the study of coordination between upper limbs is relevant for clinicians and behavioral neuroscientists. We then present a summary of how interlimb coordination is typically assessed in clinical environments and during related motor control experiments. This is followed by a proposal of categorization of interlimb tasks and different outcome measures that are applicable to each task. We believe that the growing scientific community in translational neurorehabilitation research would benefit from this condensed review.

### Why is the study of interlimb coordination relevant?

Coordination is defined in the Oxford dictionary as “the organization of the different elements of a complex body or activity so as to enable them to work together effectively” [6]. In the context of movement, motor coordination boils down to the concept of motor synergy: the very large ensemble of muscles that are activated in a cooperative way to achieve a specific motor task. The pioneering work of Bernstein [7] suggests that human motion is quite stereotyped and that motor synergy patterns are common to all humans.

More specifically, interlimb coordination refers to spatio-temporal relationships between kinematic, kinetic and physiological variables of two or more limbs executing a motor task with a common goal. Under this definition, interlimb coordination applies to tasks involving any two homologous limbs (e.g., legs during walking), two non-homologous limbs (e.g., arm and leg during dancing), or three or more limbs (e.g. legs and arms of a drummer). To narrow the scope of this paper, we will focus on interlimb coordination specifically between upper extremities, without considering the intralimb coordination between hands and fingers. This type of coordination is concerned with upper limb movements at a high level, aimed at transporting the hands to execute tasks. Although these are rather gross movements, they are commonly affected in patients with neurological impairments.

### A clinical point of view

From tying our shoes to cutting a delicious steak, proper coordination of our upper limbs is critical to our experience of everyday activities. As Johansson et al. [8] put it, the ability to coordinate two hands–and thus, both upper limbs–for effective manipulation of the environment is a hallmark of human behavior. Unfortunately, it is common that after neurological disorders, our capability to interact with the environment with grace is lost. The goal of rehabilitation clinicians is to restore this functional capability.

Rehabilitation, by definition, aims at enabling people with health conditions, experiencing or likely to experience disability, to achieve and maintain optimal functioning in interaction with the environment [9]. The integration of the arms in everyday tasks has been shown to be the single most important factor to functional upper limb recovery for people following stroke [10]. Further, upper limb dysfunction has a negative impact on ADL performance and participation of patients with multiple sclerosis [11, 12]. Thus, for a clinician, interlimb coordination is a critical feature to be restored as it affects the function of the upper limbs.

From a clinician’s point of view, the problem of coordination should be tackled in an integrated manner, rather than individually addressing separate aspects of the coordinated movement. The International Classification of Functioning, Disability and Health (ICF) from the World Health Organization (WHO) provides a framework for health and disability, dividing them into three levels: body function, activity (former ‘disability’) and participation (former ‘handicap’) [9]. Further, the ICF emphasizes the environmental factors (physical, social, attitudinal) in which people are living. According to the ICF, professionals in rehabilitation–and more specifically, neurorehabilitation–provide services (assessment and therapy) on all three ICF levels. This framework considers, for example, the important role that perception plays in the control of the complex and rich set of human voluntary movements [13], and the evidence that full potential of motor function after stroke cannot be achieved in arms with sensory deficits [14, 15]. Thus, within neurorehabilitation, clinicians consider not only motor problems (paresis, plegia, fractionated movement, coordination problems), but also abnormal muscle tone, somatosensory loss, volition, and perceptual and cognitive problems that may impact patients’ participation in society [16].

Rehabilitation professionals address patients’ needs using either a ‘top-down’ or a ‘bottom-up’ approach. The ‘top-down’ approach emphasizes the activities and participation levels of the ICF. Under this approach, clinicians try to ensure active involvement of the patient throughout the rehabilitation process and provide services toward activity limitations (difficulties a person may have in executing activities of daily living) and participation restrictions (problems an individual may experience when involved in life situations), considering environmental factors (context) that are of immediate concern to the patient [9, 17, 18]. In order to identify daily activities that have a critical influence on optimal functioning in daily life (indirectly addressing interlimb coordination problems), several client-centered interviews may be undertaken. For example, the widely used and standardized Canadian Occupational Performance Measure (COPM) [19] “is based on the premise that engagement in life roles and daily occupations of one’s own choice is a personal issue and, therefore, an important force that drives the rehabilitation process”. Thus, actual assessment of the activities of daily living (ADLs) is typically recommended. These ADL tests convey a measure of patients’ (in)dependence in ADLs (as in the widely used Barthel Index, Extended Barthel Index and several others) and/or the quality of their performance (like the Activity Analysis in terms of ADL skills or standardized Assessment of Motor and Process skills–AMPS). By applying this ‘top-down’ reasoning to assessment, clinical professionals follow the principle of client-centered practice [20]. Recently published international set of outcomes after stroke also emphasize patient-centered outcome measures, including several domains of post-stroke life, and activities like feeding, self-care, ability to return to usual daily activities, and motor functioning [21].

In contrast, the ‘bottom-up’ approach pays particular attention to the body structure and function of the ICF. Under this approach, clinicians focus on the evaluation of separate components of a patient’s skill (e.g. grasping function) and the patients’ neurological impairments [17]. Such approach supports the use of assessments that may be isolated from relevant daily life contexts, but with well-defined and standardized context (e.g. Box and Blocks test [22]). Such approach receives frequent criticism from the occupational therapists’ community given that it is not necessarily meaningful to the patient and that therapy on the underlying impairments have limited generalization into performance of daily living [23, 24].

Nevertheless, it is worth to point out that, since there are no standard assessments that objectively and quantitatively assess individual components of body function (e.g., coordination, force and impedance modulation) in the clinical practice, the cause-and-effect of impairments at this level to daily activities remains an open question. Thus, it is still premature to conclude which approach, either ‘top-down’ or ‘bottom-up’, is the most effective approach to neurorehabilitation.

### A neuroscience point of view

Neuroscience–behavioral neuroscience and motor control in particular–is focused on the determinants and regularities of normal functioning of movements. From this point of view, the interest of studying interlimb coordination is to understand how the brain controls the body’s numerous degrees-of-freedom (motor redundancy) to produce movements that are very effective and efficient in accomplishing their goals.

In contrast to the clinical approach, which mainly focuses on function and ADLs, neuroscience studies typically focus on simple, abstract tasks (e.g., finger abduction-adduction [13], wrist flexion/extension [25], circle drawing [26], point-to-point reaching [27]). Such ‘simplified’ paradigms allow neuroscientists to isolate fundamental aspects of coordination, and their link to specific brain areas such as cerebellum, supplementary motor area, cingulate motor cortex, premotor cortex, corpus callosum (see [5, 28, 29] for comprehensive reviews).

Neuroscience studies of interlimb coordination have generated different frameworks in which it can be explained and analyzed. The pioneering work of Kelso and colleagues [30–32] generated what is commonly known as dynamic-pattern theory. Under this framework, properties of interlimb coordination emerge from the self-organization of multiple sub-components. Such organization is reflected in spatiotemporal constraints upon execution of simultaneous movements. In contrast to this view, the information-processing perspective, introduced by Marteniuk et al. [33], suggests that properties of interlimb coordination are a result of "separate streams of commands that engage in neural cross-talk" and not a signature of self-organization, as proposed by the dynamic-pattern theory. In more recent years, Ivry et al. [5] have proposed a cognitive perspective. Under this framework, interlimb coordination is not only influenced by spatiotemporal constraints, but also by how tasks are cued and represented in higher centers in the brain. In this sense, properties of interlimb coordination are not only a result of motor execution, but also planning and conceptualization of the task. Through the years, ‘simplified’ paradigms used in neuroscience studies have evolved in complexity in attempts to better understand the complex phenomena of interlimb coordination in an integrated fashion, or functional unit (gestalt), rather than separate components [29].

The importance of the neuroscientific study of interlimb coordination is reflected by the significant applications it could have in the everyday clinical setting. For treatment, neuroscience studies have motivated the use of bimanual therapies (e.g. [34–37]). For assessment and diagnosis, kinematic and kinetic signatures of movement during bimanual tasks could help clinicians pinpoint deficits to specific brain areas in a non-invasive way. Such causality can be inferred from studies using transcranial magnetic stimulation or in neurologically impaired populations. For instance, Serrien et al. [38] and Steyvers et al. [39] showed how repetitive stimulation of the supplementary motor area disrupts the timing of movements; Kennerley et al. [40] documented how callosotomy patients exhibit abnormal temporal coupling (i.e., uncoupling) during a bimanual activity. Thus, specific brain areas or structures can be related to specific movement disorders.

### How is interlimb coordination currently measured?

In the previous section, we presented an overview of the diverse motivations and approaches of each field to study interlimb coordination of the upper limbs. Here, we continue with an overview of the current state-of-the-art in assessing or quantifying this type of coordination. In the clinic, protocols and outcome measures are tightly coupled, but coordination between upper limbs is rarely assessed directly. In contrast, there are various ‘classic’ neuroscience experiments that focus on interlimb coordination, but outcome measures are plentiful and less consistently used. Finally, (rehabilitation) robots are programmed by engineers to automatically compute another set of metrics that attempt to quantify coordination between the upper limbs that interact with the robot.

### Assessment of interlimb coordination by clinicians

As presented in the previous section, clinical professionals in rehabilitation are focused on returning patients’ function lost to impairments, improving their independence in the performance of activities of daily living (ADLs) and their participation in society [16]. Clinical assessments are thus focused on helping understand the source of a sensorimotor problem (diagnosis), tracking patient progress over time (monitoring), and predicting therapeutic outcomes.

Interlimb coordination and its different aspects are typically not targeted by clinical assessments, which rather measure the effects of coordination impairments on patients’ function. To this end, most clinical assessments evaluate ADLs (e.g. dressing, pouring water into a glass, picking up a coin) or closely related tasks (e.g. moving a wooden cylinder from one place to another). These assessments convey a degree of the patients’ (in)dependence in activities of daily living and/or the quality of their movement performance.

Table 1 presents commonly used clinical hand and arm assessments that contain tasks requiring coordination between upper extremities (for a detailed summary of these assessments, we recommend the reader to visit the Rehabilitation Measures Database of the Rehabilitation Institute of Chicago [41, 42]). Clinical assessments generally comprise a defined set of questions, tasks, objects, and/or instructions that are quantified according to specific scales or metrics. The meaning of the outcomes are based on validation studies of the assessment tests, which are done for each patient population the clinical test is meant to be used in. These studies generate normative data for outcome measures, as well as sensitivity, intra-and inter-rater reliability ranges, etc. The assessment administrator many times needs to be certified (and periodically re-certified) to apply the tests, to ensure strict adherence to the test protocol and scoring, and thus validity of the measurements. This is in stark contrast with the measures used in neuroscience and engineering studies, which have typically not been widely tested nor require particular administrator certification, as we will see in the next sections.

**Table 1:** Summary of clinical hand and arm assessments that require coordination between upper extremities

Assessment	Tasks involving the use of both arms	Indirect measure of interlimb coordination
Assisting Hand Assessment (AHA) [75]	22 object-related items of bimanual performance	Score and textual description
Chedoke Arm and Hand Activity Inventory (CAHAI) [76]	CAHAI-7 1. Open jar of coffee 2. Call 911 3. Draw a line with a ruler 4. Pour a glass of water 5. Wring out washcloth 6. Do up five buttons 7. Dry back with towel CAHAI-8 8. Put toothpaste on toothbrush CAHAI-9 9. Cut medium resistance putty CAHAI-13 10. Zip up zipper 11. Clean a pair of eyeglasses 12. Place container on table (8.6 kg container) 13. Carry bag up stairs (2 kg)	Score each task from 1-7, based on how independently the patient can do the task. Additional entry for which activity was done by the impaired side
The Jebsen Test of Hand Function (JTHF)-modified [77]	1 bimanual item: nut & bolt assembly	Time (speed, not quality of performance)
SHAP [78]	6 bimanual coordination items: button board, simulated food cutting, jar lid, glass jug pouring, lifting a tray, rotating a screw	Time, grip used
Purdue Pegboard Test [79]	Peg insertion with both hands simultaneously	Number of pins inserted in board in 30 s
	Nut & bolt assembly (both hands in sequence)	Number of assemblies in 1 min (right and left hands working simultaneously but each doing a separate task:get pin, put washer, put cap, put washer)
ABILHAND [80, 81]	24/26 tasks require bimanual coordination; questionnaire only	Easy/difficult/impossible
MAM-36 [82, 83]	24/36 tasks require bimanual coordination; questionnaire only	Easy/a little hard (takes long/pain/…)-very hard (can do but usually someone else will do for me)/ I can’t do/NA (did not do before injury)

Unfortunately, despite the general consensus among clinicians about the importance of standardized clinical assessments, they are not routinely performed in the clinical practice [43, 44]. Duncan et al. [43] identified four high-level determinants that impact routine assessments in practice: i) knowledge, education, and perceived value of the outcome measurement (e.g., information on validity and reliability); ii) support/priority for outcome measure use (i.e., organizational and management factors); iii) practical considerations (e.g., time, cost); iv) patient considerations (e.g., usefulness of the assessment to the patient’s treatment). Therefore, besides standardized clinical tests, it is sometimes common for clinicians to perform abstract tasks, such as reaching out to touch the clinician’s finger as it is placed in different positions in space. These simulated or contrived tasks may not be directly related to real-life situations, but they are simpler to apply. Such simplified tasks are closely related to paradigms used in motor control studies and can be easily modified to manipulate different components of interlimb coordination. In addition, we believe that the use of technology can reduce many of the burdens that prevent clinical assessments from being used on a larger scale. For instance, such simplified tasks can be easily instrumented or implemented in robotic devices used for neurorehabilitation (e.g. [45]).

While there are no clinical tests that look at coordination between arms in isolation (i.e., without the use of hands in a functional way), there are a few assessments from which interlimb coordination could be objectively assessed with the aid of technology. For instance, Inertial Motion Units (IMUs) and accelerometers are increasingly used to measure real-life performance. Such devices have been explored in healthy older persons [46, 47], post-stroke patients [48, 49], and people with Parkinsons’ disease [50, 51]. The increased capabilities and widespread availability of these technologies supports our efforts in trying to bring together different fields.

### Measurement of interlimb coordination in motor control studies

At the highest level, the execution of functional tasks depends on proper coordination of neurophysiological processes that control the involved body parts. Thus, analyzing the kinematics and kinetics of the different body parts involved in task execution gives us insight into important aspects of the mechanisms that are involved in its control. In particular, determinants of (un) healthy motor patterns can be revealed in studies of general organization laws of interlimb coordination.

Assessment of interlimb coordination of the upper limbs is challenging. As mentioned earlier, it is generally not studied in isolation, and has to be inferred from bimanual tasks [46]. Such tasks are context-dependent and have high degree of modularity [29]. Further, unlike walking, there is no general or stereotypical movement pattern; functional roles of the hands are flexible, can change across tasks [8], and dominant and non-dominant hands may perform different functions [29]. This wide range of factors complicate the quantification of movement patterns, and the generalization of results.

A complete review of studies on arm motion and interlimb coordination is beyond the scope of this paper; instead, here we report examples of paradigms and outcome measures that, in our judgement, could be easily translated into tests in a clinical setting (Table 2). The selection criteria were: i) close relatability to real-life activities, ii) suitability for widespread use, as given by the simplicity of the related set-ups, and iii) time required to perform the test.

**Table 2:** Examples of interlimb coordination-related protocols and measures used in motor control neuroscience

Paradigm	Methods	Reported measure (s) of interlimb coordination
Circle or ellipse drawing	• Trace large circles with finger tips on horizontal plane • Pacing with auditory signal • 4 conditions: both clockwise, both counter-clockwise, both inwards, and both outwards [26]	• Difference in uniformity of *relative tangential angle* • Difference in *circular variance* • Difference in *frequency deviation* • Differences in *variability of frequency* • Uniformity of *discrete relative phase* • *Aspect ratio* • Difference in *spatial variability*
	• Trace circles by moving crank arms on horizontal plane • Increasing tracing speed, from slow to fast • Distortion of visual feedback of one arm • 2 conditions: mirror symmetric starting at the same points or on opposite sides of circle [13]	• *Relative angle*
	• Trace large circles with finger tips on horizontal plane • Pacing with visual signal; 2 frequencies • 2 conditions: both inwards, and both counter-clockwise • Continuous tracing or with pause between each completed circle [84]	• Difference in uniformity of *relative tangential angle* • *Aspect ratio* • Difference in *spatial error*
Bilateral point-to-point movements	• Forward movements in horizontal plane • Targets stationary or moved when hand exceeds threshold velocity • Targets visually misrepresented closer or farther away from true target location • Gaze on non-target location [27]	• Difference in *endpoint error* • Difference in *movement duration* • Difference in *size of on-line adjustment* • Difference in *onset of on-line adjustment* • Lateral *spatial separation*
	• Forward or outward movements in horizontal plane • Targets stationary • Targets visually represented directly or through symbolic cues (i.e., letters) [56]	• Difference in *reaction time* • Difference in *movement time*
	• Draw back-and-forth lines • a vertical line task in the left limb, and a star task in the right limb (either separately or simultaneously) • Movements were restricted to the shoulder and elbow [85]	• Mean and standard deviation of orientation of each line drawing with respect to the horizontal reference position
Bilateral (physically) coupled movements	• Forward and backward movements in horizontal plane • Hands on ends of rigid bar that rotates around midpoint • Move bar without rotating [86, 87]	• *Balance error* • *Average stopping field*
	• Movements in horizontal plane • Hands on ends of stiff bar (can elongate or compress) virtually rendered between manipulandums • Transport virtual ball, that can roll along the bar, to static targets [88]	• *Absolute tilt* • Difference in *reaction time* • Change in *bar length* • Difference in *hand speed* • Difference in *hand speed peaks* • Difference in *hand path length*

### Commonly used outcome measures

As we argued in the previous section, interlimb coordination is a high-level, multi-dimensional and subjective concept. Thus, an objective assessment of interlimb coordination should include the analysis of many of the relevant features of a particular task. Specific features of kinematic, kinetic and physiological variables, captured during execution of an activity, can be used to indirectly assess interlimb coordination.

Here, we present some commonly used outcome measures across neuroscience studies.

### Relative phase

Phase measurements are commonly used for simultaneous and congruent interlimb activities, mostly in periodic tasks (for definitions see Table 4). In general, relative phase is the percent of the period that describes the lead or lag of one signal relative to the other. In most cases, this percentage is expressed in degrees (e.g. −180° corresponds to one signal leading half a period relative to the other, 0° is when the two signals match, and +180° is when the same signal lagging half a period relative the other). The relative phase measurement applied to kinematic or kinetic measurements of the limbs is interpreted as an indication of how well limbs coordinate. If the relative phase is constant and equal to zero or 180°, we can say that both limbs are moving synchronously and producing the same or the opposite movement, respectively. Likewise, variations of the relative phase over time correspond to desynchronization of the limbs.

*Relative phase* is probably the most commonly used concept in the literature. However, it should be noted that the mathematical formulation and use of the term is unsystematic, which makes the compilation of ‘normative values’–needed for standardization–difficult to achieve. Kelso and colleagues [52] measured relative phase by computing the timing of peak flexion of one limb with respect to the local peak-to-peak cycle of a metronome. Swinnen et al. [53] looked at the relative phase of a circle drawing task based on a geometric representation of the system’s state in the phase plane (position vs velocity). Mechsner et al. [13] measured the relative angle (or phase) of a circle tracing task–based on (position of the left hand vs position of the right). In contrast, Kwakkel and Wagenaar [54] computed relative phase on the phase plane defined in the acceleration-jerk space (accelerometer-based measurements). Howard et al. [55] computed relative phase between arm movements during real-life activities using a wavelet transform weighed by the cross-power of the signals (to ensure only simultaneous and congruent activities were taken into account). We note that it is important to pay attention to these differences, as people in the different fields (engineering, clinical and neuroscience) may use this terminology in very different ways and can easily lead to confusion.

### Relative reaction times and movement duration

Relative reaction time is the difference (in seconds) between the start of the movement of both limbs. Relative movement duration is the difference (in seconds) between the duration of the movement of each limb.

Reaction times are often used as indicators of spatial and temporal coupling in bimanual activities [56, 57]. Diedrichsen and Dowling [58] measured the average interval between the movement start of the left and right hands (relative reaction time) in a bimanual reaching task; the close-to-zero value obtained was interpreted as indicative of a tight temporal coupling. For back-and-forth bimanual line drawing, Franz et al. [57] computed the difference in time when the movement direction was reversed for each line segment (relative movement duration). They found out that the hands reversed direction within 10 ms of each other on at least 90 % of the movement segments, indicating temporal coupling. Similarly to the concept of relative phase, one should note that these outcome measures are ad-hoc, and comparisons of specific values from the different studies should be done with care.

### Other indirect measures

According to our definition of interlimb coordination, valid (construct validity) measures should analyze spatio-temporal relationships between the kinematic, kinetic and physiological variables of the limbs involved in the task. However, if the goal of a bimanual task is to involve coordination of the two limbs, it may still be valid to use task performance measures or compute relationships against an equivalent unimanual task as indirect indicators of interlimb coordination.

For example, Lewis and Perreault [59] compared muscular activity from robot-assisted unimanual and bimanual tasks. Authors recorded electromyography (EMG) from the anterior deltoid, middle deltoid, biceps brachii, and the lateral head of the triceps brachii muscles. The onset of muscular activity was determined as the first point to increase above three standard deviations of the pre-movement mean EMG activity in the same profile. The peak of EMG activity was determined as an indicator of the extent of muscle activation. From these, researchers looked at the *relative timing of muscular activity onset*, *relative timing of peak muscular activity* and *relative timing of peak force* between unimanual and bimanual movement conditions to investigate coordination.

### Assessment of interlimb coordination by state-of-the-art rehabilitation devices - the engineering approach

Over the past years, robotic devices are being increasingly used to assess sensorimotor behavior [45]. Balasubramanian et al. [60] and Nordin et al. [61] presented comprehensive reviews of different movement quality measures that have been most frequently described in the rehabilitation robotics literature. Nordin et al. [61] categorized the measures: variability, spatial contraction/expansion, systematic shifts (from [62]), matching position error, medial/lateral shift & skew, anterior/posterior shift & skew, and shrink coefficient (from [63]) as measures of interlimb coordination. However, we should note that such measures are not representative of interlimb coordination; such measures were designed to assess limb position sense in arm-matching tasks with one arm active and the other arm passive. As the two limbs are not both actively involved, it is not accurate to say that these measures relate to interlimb coordination according to our definition.

van Delden et al. [64] presented a systematic review of bilateral upper limb devices that have been developed since 1990. Out of 311 single citations on bilateral upper limb training, they identified 20 different bilateral training devices, both mechanical and robotic. Here, we extended this list by adding a few other devices and the reported measures of interlimb coordination and interlimb activities that they enable (Table 3). Interestingly, most bilateral training devices to date do not provide tools for assessing interlimb coordination, even though the typical motivation to develop such devices is that interlimb coordination is fundamental for functioning in everyday life. Additionally, the few measures offered by some of the devices are not directly comparable to the measures used by either clinicians or neuroscientists (note the contrast to sections 3.1 and 3.2, respectively).

**Table 3:** Summary of bilateral training devices and reported measures of interlimb coordination

Device	Interlimb activities allowed by the device	Reported measure of interlimb coordination
Able-X [89]	Coupled arm movements in free space.	None
Adaptive Bimanual Robotic Training [90]	Coupled, visual and point symmetric.	• *Relative power* between arms • *Rotational error*
APBT (the Rocker) [91, 92]	Mirror or near-symmetric (phase lag of 60°) wrist flexion and extension movements in the horizontal plane.	None
ARCMIME [93]	Uncoupled arm movements in one DOF (forwards and backwards). Adjustable plane.	None
Batrac [94–96]	Uncoupled arm movements in one DOF (forwards and backwards). Adjustable plane.	None
BFIAMT [97]	Forward and backward movement over parallel tracks, bilateral reciprocal, and bilateral symmetric upper limb movement.	None
Bimanual Handlebar [98]	Coupled forward and backward arm movements as well as rotational movements of the end-effector around an axis perpendicular to the direction of the translation.	*Rotational error*
Bimanual-Coordinated Training System [99]	Uncoupled movements, one DOF.	None
Bi-Manu-Track [100, 101]	Mirror and visual symmetric forearm pronosupination and wrist flexion/extension.	None
Braccio Di Ferro [86, 87, 102]	Any, constrained to planar movements of the arm in the transverse plane involving elbow and shoulder flexion/extension.	*Average stopping field*; *balance error* during a coupled reaching task
Driver’s SEAT [103, 104]	Coupled, point-symmetric movements.	None
EXO-UL7 [105, 106]	Any, free space.	*Efficiency index* for the Bilateral Movement Training
Hand Robotic Rehabilitation Device [107]	Forearm pronosupination and wrist flexion/extension movements.	None
Hand-Object-Hand (H-O-H) [108]	Coupled wrist flexion/extension movements.	None
KINARM [88]	Any, constrained to planar movements of the arm in the transverse plane involving elbow and shoulder flexion/extension.	• *Absolute tilt* • Difference in *reaction time* • *Change in bar length* • Difference in *hand speed* • Difference in *hand speed peaks* • Difference in *hand path length*
MIME [109–112]	Any arm movement.	None
Reha-Slide [113]	Coupled arm movements in one DOF (forwards and backwards). Adjustable plane.	None
Reha-Slide Duo [114]	Uncoupled arm movements in one DOF (forwards and backwards). Adjustable plane.	None
The Bimanual Lifting Rehabilitator [115]	Coupled arm movements, similar to lifting a tray.	*Time series similarity* between right and left hand lift forces
Virtual Reality Piano [116]	Any.	• *Performance time* • *Sequential accuracy*

### Towards a common language on the assessment of interlimb coordination

As the reader may have already concluded, the assessment of interlimb coordination is far from being systematic – not only across fields, but also within fields. We believe that, in order to move forward, we first need to standardize the way interlimb activities are described. Standardization of protocols and measures at this point is difficult because the scientific literature is full of ambiguous definitions of an observed action or phenomenon that is usually only valid within a specific study. For example, the term ‘symmetric task’ has been used to describe a task with perceptual or visual symmetry, but that requires the use of non-homologous muscles, and also to describe a task in which homologous muscles are used [25]. This ambiguity and lack of consistency in terminology prevents (or slows down) the collection of normative data that can be used in the neuro-rehabilitation community. Therefore, we believe that before diving into the standardization of outcome measures and protocols, we first need to contextualize the tasks. This should help identify which of the terms are relevant to the task being studied. For example, many neuroscience studies focus on isometric tasks–i.e., without movement–for which spatial descriptors are meaningless (unless the task involves e.g., a virtual display). Further, tasks can be active or passive. In the time domain, we differentiate between discrete and continuous tasks according to whether they have definite start and end points related to the goal of the task, e.g., reaching out to a fixed target (discrete) or tracking a moving target (continuous). Of further interest is to distinguish between periodic and non-periodic tasks, according to whether they are cyclic or not.

To our knowledge, the only attempt to define a comprehensive taxonomy of interlimb activities, in particular bimanual tasks, was 30 years ago by MacKenzie and Marteniuk [65]. Since then, definitions have slowly fallen out of use. In order to standardize the scientific jargon, we propose a categorization (taxonomy) of interlimb activities (Table 4). This categorization is not meant to be exhaustive, and is based on commonly used terms from the recent literature, in particular, from the ones reported in this paper. The taxonomy focuses on upper limb tasks, although many of these definitions could also be applied to other interlimb relationships.

**Table 4:** Categorization of interlimb tasks

Category	Domain	Short description	Examples
Adjectives that describe the actions of one limb independent of the other			
Discrete/Continuous	Temporal	Discrete tasks involve actions with a definite beginning and end [117]. Conversely, continuous tasks involve actions that lack such recognizable events [118, 119].	Discrete: point-to-point reaching movements, pushing a button. Continuous: tracking a moving target, steering a driving wheel.
Periodic/Non-periodic	Spatio-temporal	Periodic tasks are those in which a particular movement is repeated at (quasi-) regular intervals. In non-periodic tasks, the intervals are not regular or the action does not repeat.	Periodic: drawing multiple circles at the same frequency without stopping. Non-periodic: drawing one circle or drawing multiple circles of increasing radius at the same frequency without stopping.
Isometric/Non-isometric	Spatial	Isometric tasks do not involve limb movement, but require the production of forces. Non-isometric tasks require movement.	Isometric: pushing a wall that does not move. Non-isometric: moving an object.
Active/Passive	Movement execution	In an active task, the user is required to perform or try to perform a specific action. In a passive task, the user’s limb is moved by an external agent (e.g., a robot) and the user is instructed to relax and not interfere with the movement.	Active: move your arm by contracting your muscles. Passive: a robot to moves your arm without you trying to interfere.
Adjectives that describe the actions of the two limbs relative to each other			
Simultaneous/Sequential	Temporal	If limbs execute actions at the same time, the task is simultaneous. If the action of one limb ends and is followed by the action of the other limb, it is a sequential task. We note that the terms ‘synchronous’ and ‘asynchronous’ have been used as substitutes for simultaneous/sequential. However, we discourage their use, as they can be confused with in-/out-of-phase (see below).	Simultaneous: cutting a steak. Sequential: opening a drawer with one hand and retrieving objects from inside the drawer with the other hand after the drawer is opened.
In-phase/Out-of-phase	Temporal	This adjective is relative to the start of the movement For periodic tasks or single cycles of movement, a task is in-phase if the relative phase between the movements of the limbs is zero. In out-of-phase tasks, the relative phase is non-zero. Note that these categories also apply to movements in which the frequency of one limb is a harmonic of the other, as there is a minimum frequency that is common to both. This common frequency is used as reference to determine the in-/out-of-phase characteristic of the task.	In-phase: arm movements during breast-stroke in swimming; drummer that does one beat with one hand while doing two with the other. Out-of-phase: arm movements during front crawl in swimming; drawing ellipses starting from different points on the perimeter.
Mirror symmetric/Visual symmetric/Point symmetric	Spatio-temporal	Within the neuroscience community, the term *symmetric* has been used to tasks in which homologous muscles are used and *asymmetric* to tasks in which non-homologous muscles are used. However, such convention is unintuitive for someone unfamiliar with the historical background of the study of bimanual coordination. Therefore, we suggest to define symmetric tasks as proposed by Malabet et al. [35]: • Mirror symmetric-the movement of one limb reflects the movements of the other as if a mirror were placed in the mid-sagittal plane. • Visual symmetric-the endpoints of the two limbs move in the same direction and with the same magnitude (this is also referred as asymmetric in the literature; however, we discourage this use as it can be confused with incongruent movements). • Point symmetric-the movement of the limbs are opposed relative to a point in space. To avoid confusion with previous literature, the term *incongruent* should be used for tasks in which limbs perform asymmetric movements (i.e. that cannot be categorized as mirror, visual or point symmetric).	Mirror symmetric: jumping jacks. Visual symmetric: moving a tray with two hands forward and backward, and left to right (without rotations). Point symmetric: turning a steering wheel with hands on opposite sides of a diameter (180° apart).
Congruent/Incongruent	Spatio-temporal	Congruent tasks are symmetric and in-phase. Incongruent tasks are those in which the task assigned to each limb differs with respect to a least one parameter [5].	Congruent: drawing two ‘identical’ circles at the same time. Incongruent: drawing a circle with one hand and a line with the other; drawing circles with different radii with each hand.
(Physically) Coupled/Uncoupled	Spatial	Coupled tasks are those in which the limbs are mechanically or virtually (external to the body) connected. In order to be considered coupled, the connection should allow one limb to have an effect on the dynamics of the opposite limb. During uncoupled tasks, limbs move independently (e.g. [120]).	Coupled: turning a steering wheel, sweeping, squeezing a rubber ball with both hands. Uncoupled: drawing a circle in the air with each hand.

### Recommendations for future technology-aided assessments

#### On the task and protocols

Given the significant amount of neuroscience research in the field of interlimb coordination, we suggest introducing simple tests taken from neuroscience studies in the clinical practice. Although some of these protocols are abstract and not related to ADLs, one could relate the different outcome measures to brain studies.

#### On the use of mechanical or robotic training devices

It is critical that the device not interfere with the movement. Factors that can bias measurements of interlimb coordination include, using devices with different mechanical properties (i.e. friction, inertia) and improper calibration of sensors (force sensors, in particular).

#### On task instructions and presentation of information

Interlimb coordination depends on how actions are represented on a cognitive level [5]. Such action representations are highly affected by the task instructions and what/how information is presented during the task. For example, in a simultaneous periodic task, Bogaerts et al. [66] asked subjects to draw lines back and forth, while manipulating visual information of the trajectories on a screen. They observed that the accuracy and stability of coordination patterns were affected by the display of the correct or a transformed version of the trajectories. In another task, Lee et al. [67] compared the coordination of two groups trying to perform a simultaneous, out-of-phase, uncoupled, periodic task at different frequencies. One group received the instruction to “not intervene” when they felt themselves slipping out of the anti-phase pattern, while the other group was told to “try to stay with the pattern” at all times. The “not intervene” group replicated previous findings of phase switching with increasing frequencies, while the other group showed very different results.

The modality of information used for feedback is also relevant to the way we encode information. In the experiment presented by Ronsse et al. [68], authors showed how learning a bimanual task with visual feedback–reflecting coordination between hands–differed to auditory feedback–reflecting an integrating timing of both hands. In their study, Ronsse et al. [68] found differences in brain activity, feedback dependency and performance after learning.

Our recommendation is to always rely on scripted task instructions when communicating with experimental subjects. To improve future standardization, scripted tasks instructions and screenshots of the visual feedback used should be included as supplementary materials when publishing manuscripts.

#### On the cognitive load demanded by the task

The use of hands in daily life can be compromised by additional cognitive load. When a verbal-cognitive task was added to a movement task, dual-task interference occurred in a group of unimpaired subjects, and to an even greater extent in people with mild-to-moderate Parkinson’s disease [69]. Thus, coordination assessments should minimize concurrent cognitive tasks and stimuli.

#### On the use of virtual environments

Many of the aforementioned assessment tasks, including clinical assessments, could be done in a virtual environment. For example, Lambercy et al. [70] used a virtual implementation of the Peg Insertion Test to assess upper limb motor function in patients with multiple sclerosis. However, how feedback is rendered to the subject should be carefully considered. For example, differences in motor task performance have been observed between setups using, e.g., horizontal or vertical displays [71, 72], and different brain areas can be activated depending on the feedback provided, e.g. [68]. Further immersion in virtual reality could better emulate visual feedback, for example using head mounted displays such as the Oculus Rift. However, care should be taken to avoid or address the distorted perception of 3D space that arises in such systems [73]. An alternative solution is represented by exer-games, in which patients are guided through graphics to execute specific exercises. Moreover, it provides the feedback element clearly identified as fundamental to learning and to produce an ecological setting [74]. This approach has revealed powerful to boost motivation and it could be worthwhile to extend to assessments [73].

## Conclusions

Our long-term goal is to standardize the use of robotic-and sensor-based assessments. Our objective is to work towards a unified framework for the assessment of interlimb coordination in the clinical practice with sound foundations on neuroscience studies. However, defining what to measure can currently be very frustrating, as the definition and mathematical algorithms of terms commonly used to describe interlimb coordination (e.g. relative phase, symmetry, etc) vary considerably across the scientific literature. In addition, there are no universal measures to quantify coordination of interlimb tasks, as many measures are ad-hoc and restricted to very specific scenarios and protocols, with limited transferability to the clinical practice.

Here, we presented a general definition of interlimb coordination and its relevance from the clinical and neuroscience perspectives. A general taxonomy of interlimb activities and a review on the different approaches to assess interlimb coordination was also presented. Throughout this paper, we tried to show that, despite our implicit understanding of the concept of coordination, it is a complex phenomenon that cannot be quantified with a single parameter. At the higher level, clinicians and neuroscientists agree on the importance of interlimb coordination, given its relationship to movement dysfunctions. However, large incongruences on the specific measures to assess interlimb coordination reflects the different interpretations/points of view at the lower level.

This paper represents a first step towards standardization of the jargon and vocabulary used in interlimb coordination across the scientific community. We hope that these efforts will prompt the scientific community to unify findings and facilitate the standardization of related assessment protocols. We hope that this will drive further work into the collection of normative and representative data.

## Abbreviations

ADL: Activities of daily living
AHA: Assisting hand assessment
CAHAI: Chedoke arm and hand activity inventory
ICF: International classification of functioning, disability and health
JTHF: Jebsen test of hand function
WHO: World health organization

## References

[1] Keller T. 2011. COST Action TD1006. http://www.cost.eu/COST_Actions/bmbs/TD1006. Accessed Jan 2016.

[2] Johansson RS, Westling G, Backstrom A, Flanagan JR (2001). Eye-hand coordination in object manipulation. J Neurosci.

[3] Borghese NA, Bianchi L, Lacquaniti F (1996). Kinematic determinants of human locomotion. J Physiol.

[4] Cirstea MC, Mitnitski AB, Feldman AG, Levin MF (2003). Interjoint coordination dynamics during reaching in stroke. Exp Brain Res.

[5] Ivry R, Diedrichsen J, Spencer R, Hazeltine E, Semjen A, Swinnen S, Duysens J (2004). A Cognitive Neuroscience Perspective on Bimanual Coordination and Interference. Neuro-Behavioral Determinants of Interlimb Coordination. Springer US.

[6] Stevenson A (2010). Oxford dictionary of English.

[7] Bernstein NA (1967). The co-ordination and regulation of movements.

[8] Johansson RS, Theorin A, Westling G, Andersson M, Ohki Y, Nyberg L (2006). How a Lateralized Brain Supports Symmetrical Bimanual Tasks. PLoS Biol.

[9] Stucki G, Cieza A, Melvin J (2007). The International Classification of Functioning, Disability and Health (ICF): a unifying model for the conceptual description of the rehabilitation strategy. J Rehabil Med.

[10] Barker RN, Gill TJ, Brauer SG (2007). Factors contributing to upper limb recovery after stroke: a survey of stroke survivors in Queensland Australia. Disabil Rehabil.

[11] Kierkegaard M, Einarsson U, Gottberg K, Von Koch L, Holmqvist LW (2012). The relationship between walking, manual dexterity, cognition and activity/participation in persons with multiple sclerosis. Mult Scler.

[12] Yozbatiran N, Baskurt F, Baskurt Z, Ozakbas S, Idiman E (2006). Motor assessment of upper extremity function and its relation with fatigue, cognitive function and quality of life in multiple sclerosis patients. J Neurol Sci.

[13] Mechsner F, Kerzel D, Knoblich G, Prinz W (2001). Perceptual basis of bimanual coordination. Nature.

[14] Brasil-Neto JP, De Lima AC (2008). Sensory deficits in the unaffected hand of hemiparetic stroke patients. Cogn Behav Neurol.

[15] Dannenbaum RM, Dykes RW (1988). Sensory loss in the hand after sensory stroke: therapeutic rationale. Arch Phys Med Rehabil.

[16] WHO (2001). World Health Organization, International classification of functioning, disability and health.

[17] Fisher AG (2009). Occupational therapy intervention process model: a model for planning and implementing top-down, client-centered and occupation-based interventions.

[18] Neistadt ME (2000). Occupational therapy evaluation for adults: a pocket guide.

[19] Law M, Baptiste S, McColl M, Opzoomer A, Polatajko H, Pollock N (1990). The Canadian occupational performance measure: an outcome measure for occupational therapy. Can J Occup Ther.

[20] Rogers CR (1966). Client-centered therapy.

[21] Salinas J, Sprinkhuizen SM, Ackerson T, Bernhardt J, Davie C, George MG, Gething S, Kelly AG, Lindsay P, Liu L, Martins SC, Morgan L, Norrving B, Ribbers GM, Silver FL, Smith EE, Williams LS, Schwamm LH (2016). An International Standard Set of Patient-Centered Outcome Measures After Stroke. Stroke.

[22] Mathiowetz V, Volland G, Kashman N, Weber K (1985). Adult norms for the Box and Block Test of manual dexterity. Am J Occup Ther.

[23] Bernspang B, Asplund K, Eriksson S, Fugl-Meyer AR (1987). Motor and perceptual impairments in acute stroke patients: effects on self-care ability. Stroke.

[24] Jongbloed L, Brighton C, Stacey S (1988). Factors associated with independent meal preparation, self-care and mobility in CVA clients. Can J Occup Ther.

[25] Kelso JA (1984). Phase transitions and critical behavior in human bimanual coordination. Am J Physiol.

[26] Carson RG, Thomas J, Summers JJ, Walters MR, Semjen A (1997). The dynamics of bimanual circle drawing. Q J Exp Psychol A.

[27] Diedrichsen J, Nambisan R, Kennerley SW, Ivry RB (2004). Independent on-line control of the two hands during bimanual reaching. Eur J Neurosci.

[28] Swinnen SP (2002). Intermanual coordination: from behavioural principles to neural-network interactions. Nat Rev Neurosci.

[29] Swinnen SP, Wenderoth N (2004). Two hands, one brain: cognitive neuroscience of bimanual skill. Trends Cogn Sci.

[30] Haken H, Kelso JA, Bunz H (1985). A theoretical model of phase transitions in human hand movements. Biol Cybern.

[31] Kelso JA, Southard DL, Goodman D (1979). On the nature of human interlimb coordination. Science.

[32] Kelso JAS (1995). Dynamic patterns: the self-organization of brain and behavior.

[33] Marteniuk RG, Mackenzie CL, Baba DM (1984). Bimanual Movement Control-Information-Processing and Interaction Effects. Q J Exp Psychol A Hum Exp Psychol.

[34] Lum PS, Reinkensmeyer DJ, Lehman SL (1993). Robotic assist devices for bimanual physical therapy: preliminary experiments. IEEE Trans Rehabil Eng.

[35] Malabet HG, Robles RA, Reed KB (2010). Symmetric Motions for Bimanual Rehabilitation. Ieee/Rsj 2010 International Conference on Intelligent Robots and Systems (Iros 2010).

[36] Rose DK, Winstein CJ (2004). Bimanual training after stroke: Are two hands better than one?. Top Stroke Rehabil.

[37] Sleimen-Malkoun R, Temprado J-J, Thefenne L, Berton E (2011). Bimanual training in stroke: How do coupling and symmetry-breaking matter?. BMC Neurol.

[38] Serrien DJ, Strens LH, Oliviero A, Brown P (2002). Repetitive transcranial magnetic stimulation of the supplementary motor area (SMA) degrades bimanual movement control in humans. Neurosci Lett.

[39] Steyvers M, Etoh S, Sauner D, Levin O, Siebner HR, Swinnen SP, Rothwell JC (2003). High-frequency transcranial magnetic stimulation of the supplementary motor area reduces bimanual coupling during anti-phase but not in-phase movements. Exp Brain Res.

[40] Kennerley SW, Diedrichsen J, Hazeltine E, Semjen A, Ivry RB (2002). Callosotomy patients exhibit temporal uncoupling during continuous bimanual movements. Nat Neurosci.

[41] Center for Rehabilitation Outcomes Research (CROR), Dept. of Medical Social Sciences Informatics Group, N.U., Rehabilitation Measures Database. www.rehabmeasures.org. Accessed 20 May 2015.

[42] Moore JL, Raad J, Ehrlich-Jones L, Heinemann AW (2014). Development and use of a knowledge translation tool: the rehabilitation measures database. Arch Phys Med Rehabil.

[43] Duncan E, Murray J (2012). The barriers and facilitators to routine outcome measurement by allied health professionals in practice: a systematic review. BMC Health Serv Res.

[44] Jette DU, Halbert J, Iverson C, Miceli E, Shah P (2009). Use of Standardized Outcome Measures in Physical Therapist Practice: Perceptions and Applications. Phys Ther.

[45] Scott SH, Dukelow SP (2011). Potential of robots as next-generation technology for clinical assessment of neurological disorders and upper-limb therapy. J Rehabil Res Dev.

[46] Kilbreath SL, Heard RC (2005). Frequency of hand use in healthy older persons. Aust J Physiother.

[47] Rand D, Eng JJ (2010). Arm-Hand Use in Healthy Older Adults. Am J Occup Ther.

[48] Gebruers N, Vanroy C, Truijen S, Engelborghs S, De Deyn PP (2010). Monitoring of physical activity after stroke: a systematic review of accelerometry-based measures. Arch Phys Med Rehabil.

[49] Uswatte G, Miltner WH, Foo B, Varma M, Moran S, Taub E (2000). Objective measurement of functional upper-extremity movement using accelerometer recordings transformed with a threshold filter. Stroke.

[50] Horne MK, McGregor S, Bergquist F (2015). An objective fluctuation score for Parkinson’s disease. PLoS ONE.

[51] LeMoyne R, Mastroianni T, Grundfest W (2013). Wireless accelerometer configuration for monitoring Parkinson’s disease hand tremor.

[52] Kelso JAS, Delcolle JD, Schoner G (1990). Action-Perception as a Pattern-Formation Process. Attention and Performance Xiii.

[53] Swinnen SP, Jardin K, Meulenbroek R (1996). Between-limb asynchronies during bimanual coordination: Effects of manual dominance and attentional cueing. Neuropsychologia.

[54] Kwakkel G, Wagenaar RC (2002). Effect of duration of upper-and lower-extremity rehabilitation sessions and walking speed on recovery of interlimb coordination in hemiplegic gait. Phys Ther.

[55] Howard IS, Ingram JN, Körding KP, Wolpert DM (2009). Statistics of Natural Movements Are Reflected in Motor Errors. J Neurophysiol.

[56] Diedrichsen J, Hazeltine E, Kennerley S, Ivry RB (2001). Moving to directly cued locations abolishes spatial interference during bimanual actions. Psychol Sci.

[57] Franz EA, Eliassen JC, Ivry RB, Gazzaniga MS (1996). Dissociation of spatial and temporal coupling in the bimanual movements of callosotomy patients. Psychol Sci.

[58] Diedrichsen J, Dowling N (2009). Bimanual coordination as task-dependent linear control policies. Hum Mov Sci.

[59] Lewis GN, Perreault EJ (2009). An Assessment of Robot-Assisted Bimanual Movements on Upper Limb Motor Coordination Following Stroke. IEEE Trans Neural Syst Rehabil Eng.

[60] Balasubramanian S, Colombo R, Sterpi I, Sanguineti V, Burdet E (2012). Robotic assessment of upper limb motor function after stroke. Am J Phys Med Rehabil.

[61] Nordin N, Xie SQ, Wunsche B (2014). Assessment of movement quality in robot-assisted upper limb rehabilitation after stroke: a review. J Neuroeng Rehabil.

[62] Dukelow SP, Herter TM, Moore KD, Demers MJ, Glasgow JI, Bagg SD, Norman KE, Scott SH (2010). Quantitative assessment of limb position sense following stroke. Neurorehabil Neural Repair.

[63] Squeri V, Zenzeri J, Morasso P, Basteris A (2011). Integrating proprioceptive assessment with proprioceptive training of stroke patients. IEEE Int Conf Rehabil Robot.

[64] Van Delden AL, Peper CL, Kwakkel G, Beek PJ (2012). A systematic review of bilateral upper limb training devices for poststroke rehabilitation. Stroke Res Treat.

[65] MacKenzie CL, Marteniuk RG, Eric AR (1985). Bimanual Coordination.

[66] Bogaerts H, Buekers MJ, Zaal FT, Swinnen SP (2003). When visuo-motor incongruence aids motor performance: the effect of perceiving motion structures during transformed visual feedback on bimanual coordination. Behav Brain Res.

[67] Lee TD, Blandin Y, Proteau L (1996). Effects of task instructions and oscillation frequency on bimanual coordination. Psychol Res.

[68] Ronsse R, Puttemans V, Coxon JP, Goble DJ, Wagemans J, Wenderoth N, Swinnen SP (2011). Motor learning with augmented feedback: modality-dependent behavioral and neural consequences. Cereb Cortex.

[69] Proud EL, Morris ME (2010). Skilled hand dexterity in Parkinson’s disease: effects of adding a concurrent task. Arch Phys Med Rehabil.

[70] Lambercy O, Fluet MC, Lamers I, Kerkhofs L, Feys P, Gassert R (2013). Assessment of upper limb motor function in patients with multiple sclerosis using the Virtual Peg Insertion Test: a pilot study. IEEE Int Conf Rehabil Robot.

[71] Parmar PN, Huang FC, Patton JL (2011). Simultaneous coordinate representations are influenced by visual feedback in a motor learning task. Conf Proc IEEE Eng Med Biol Soc.

[72] Parmar PN, Huang FC, Patton JL (2015). Evidence of multiple coordinate representations during generalization of motor learning. Exp Brain Res.

[73] Kelly JW, Burton M, Pollock B, Rubio E, Curtis M, Cruz JDL, Gilbert S, Winer E (2013). Space perception in virtual environments: Displacement from the center of projection causes less distortion than predicted by cue-based models. ACM Trans Appl Percept.

[74] Pirovano M, Mainetti R, Baud-Bovy G, Lanzi PL, Borghese NA (2016). Intelligent Game Engine for Rehabilitation (IGER). IEEE Trans Comput Intell AI Games.

[75] Krumlinde-Sundholm L, Eliasson A-C (2003). Development of the Assisting Hand Assessment: a Rasch-built measure intended for children with unilateral upper limb impairments. Scand J Occup Ther.

[76] Barreca S, Gowland C, Stratford P, Huijbregts M, Griffiths J, Torresin W, Dunkley M, Miller P, Masters L (2004). Development of the Chedoke Arm and Hand Activity Inventory: theoretical constructs, item generation, and selection. Top Stroke Rehabil.

[77] Jones L, Harrison J (1987). Evaluation of hand movements used during hand testing and activities of daily living. Int J Rehabil Res.

[78] Light CM, Chappell PH, Kyberd PJ (2002). Establishing a standardized clinical assessment tool of pathologic and prosthetic hand function: normative data, reliability, and validity. Arch Phys Med Rehabil.

[79] Tiffin J (1948). Purdue pegboard test. Chicago: Science Research 194.

[80] Barrett LE, Cano SJ, Zajicek JP, Hobart JC (2013). Can the ABILHAND handle manual ability in MS?. Mult Scler.

[81] Penta M, Thonnard JL, Tesio L (1998). ABILHAND: a Rasch-built measure of manual ability. Arch Phys Med Rehabil.

[82] Chen CC, Bode RK (2010). Psychometric validation of the Manual Ability Measure-36 (MAM-36) in patients with neurologic and musculoskeletal disorders. Arch Phys Med Rehabil.

[83] Chen CC, Palmon O, Amini D (2014). Responsiveness of the Manual Ability Measure-36 (MAM-36): changes in hand function using self-reported and clinician-rated assessments. Am J Occup Ther.

[84] Summers JJ, Maeder S, Hiraga CY, Alexander JR (2008). Coordination dynamics and attentional costs of continuous and discontinuous bimanual circle drawing movements. Hum Mov Sci.

[85] Swinnen SP, Puttemans V, Vangheluwe S, Wenderoth N, Levin O, Dounskaia N (2003). Directional interference during bimanual coordination: is interlimb coupling mediated by afferent or efferent processes. Behav Brain Res.

[86] Sanguineti V, Casadio M, Vergaro E, Squeri V, Giannoni P, Morasso PG (2009). Robot therapy for stroke survivors: proprioceptive training and regulation of assistance. Stud Health Technol Inform.

[87] Squeri V, Casadio M, Vergaro E, Giannoni P, Morasso P, Sanguineti V (2009). Bilateral robot therapy based on haptics and reinforcement learning: Feasibility study of a new concept for treatment of patients after stroke. J Rehabil Med.

[88] Lowrey C, Jackson C, Bagg S, Dukelow S, Scott S (2014). A Novel Robotic Task for Assessing Impairments in Bimanual Coordination Post-Stroke. Int J Phys Med Rehabil S.

[89] Hijmans JM, Hale LA, Satherley JA, McMillan NJ, King MJ (2011). Bilateral upper-limb rehabilitation after stroke using a movement-based game controller. J Rehabil Res Dev.

[90] Trlep M, Mihelj M, Puh U, Munih M (2011). Rehabilitation Robot with Patient-Cooperative Control for Bimanual Training of Hemiparetic Subjects. Adv Robot.

[91] Stinear CM, Barber PA, Coxon JP, Fleming MK, Byblow WD (2008). Priming the motor system enhances the effects of upper limb therapy in chronic stroke. Brain.

[92] Stinear JW, Byblow WD (2004). Rhythmic bilateral movement training modulates corticomotor excitability and enhances upper limb motricity poststroke: a pilot study. J Clin Neurophysiol.

[93] Mahoney RM, Van der Loos HFM, Lum PS, Burgar C (2003). Robotic stroke therapy assistant. Robotica.

[94] Richards LG, Senesac CR, Davis SB, Woodbury ML, Nadeau SE (2008). Bilateral arm training with rhythmic auditory cueing in chronic stroke: not always efficacious. Neurorehabil Neural Repair.

[95] Whitall J, McCombe Waller S, Silver KH, Macko RF (2000). Repetitive bilateral arm training with rhythmic auditory cueing improves motor function in chronic hemiparetic stroke. Stroke.

[96] Whitall J, Waller SM, Sorkin JD, Forrester LW, Macko RF, Hanley DF, Goldberg AP, Luft A (2011). Bilateral and unilateral arm training improve motor function through differing neuroplastic mechanisms: a single-blinded randomized controlled trial. Neurorehabil Neural Repair.

[97] Chang JJ, Tung WL, Wu WL, Huang MH, Su FC (2007). Effects of robot-aided bilateral force-induced isokinetic arm training combined with conventional rehabilitation on arm motor function in patients with chronic stroke. Arch Phys Med Rehabil.

[98] Trlep M, Mihelj M, Munih M (2012). Skill transfer from symmetric and asymmetric bimanual training using a robotic system to single limb performance. J Neuroeng Rehabil.

[99] Li CG, Inoue Y, Liu T, Shibata K, Oka K (2011). A New Master-Slave Control Method for Implementing Force Sensing and Energy Recycling in a Bilateral Arm Training Robot. Int J Innov Comput Inf Control.

[100] Hesse S, Schulte-Tigges G, Konrad M, Bardeleben A, Werner C (2003). Robot-assisted arm trainer for the passive and active practice of bilateral forearm and wrist movements in hemiparetic subjects. Arch Phys Med Rehabil.

[101] Hesse S, Werner C, Pohl M, Rueckriem S, Mehrholz J, Lingnau ML (2005). Computerized arm training improves the motor control of the severely affected arm after stroke: a single-blinded randomized trial in two centers. Stroke.

[102] Casadio M, Sanguineti V, Morasso PG, Arrichiello V (2006). Braccio di Ferro: A new haptic workstation for neuromotor rehabilitation. Technol Health Care.

[103] Johnson MJ, Van der Loos HFM, Burgar CG, Shor P, Leifer LJ (2003). Design and evaluation of Driver’s SEAT: A car steering simulation environment for upper limb stroke therapy. Robotica.

[104] Johnson MJ, Van der Loos HFM, Burgar CG, Shor P, Leifer LJ (2005). Experimental results using force-feedback cueing in robot-assisted stroke therapy. IEEE Trans Neural Syst Rehabil Eng.

[105] Kim H, Miller LM, Fedulow I, Simkins M, Abrams GM, Byl N, Rosen J (2013). Kinematic Data Analysis for Post-Stroke Patients Following Bilateral Versus Unilateral Rehabilitation With an Upper Limb Wearable Robotic System. IEEE Trans Neural Syst Rehabil Eng.

[106] Rosen J, Perry JC (2007). Upper limb powered exoskeleton. Int J Humanoid Rob.

[107] Rashedi E, Mirbagheri A, Taheri B, Farahmand F, Vossoughi GR, Parnianpour M (2009). Design and development of a hand robotic rehabilitation device for post stroke patients. Conf Proc IEEE Eng Med Biol Soc.

[108] Lum PS, Reinkensmeyer DJ, Lehman HL, Szeto Andrew YJ, Rangayyan Rangaraj M (1993). Bimanual reflex during two hand grasp.

[109] Burgar CG, Lum PS, Scremin AM, Garber SL, Van der Loos HF, Kenney D, Shor P (2011). Robot-assisted upper-limb therapy in acute rehabilitation setting following stroke: Department of Veterans Affairs multisite clinical trial. J Rehabil Res Dev.

[110] Burgar CG, Lum PS, Shor PC, Machiel Van der Loos HF (2000). Development of robots for rehabilitation therapy: the Palo Alto VA/Stanford experience. J Rehabil Res Dev.

[111] Lum PS, Burgar CG, Shor PC, Majmundar M, Van der Loos M (2002). Robot-assisted movement training compared with conventional therapy techniques for the rehabilitation of upper-limb motor function after stroke. Arch Phys Med Rehabil.

[112] Lum PS, Burgar CG, Van der Loos M, Shor PC, Majmundar M, Yap R (2006). MIME robotic device for upper-limb neurorehabilitation in subacute stroke subjects: A follow-up study. J Rehabil Res Dev.

[113] Hesse S, Schmidt H, Werner C, Rybski C, Puzich U, Bardeleben A (2007). A new mechanical arm trainer to intensify the upper limb rehabilitation of severely affected patients after stroke: design, concept and first case series. Eura Medicophys.

[114] Buschfort R, Brocke J, Hess A, Werner C, Waldner A, Hesse S (2010). Arm studio to intensify the upper limb rehabilitation after stroke: concept, acceptance, utilization and preliminary clinical results. J Rehabil Med.

[115] Lum SP, Lehman SL, Reinkensmeyer DJ (1995). The bimanual lifting rehabilitator: an adaptive machine for therapy of stroke patients. IEEE Trans Neural Syst Rehabil Eng.

[116] Adamovich SV, Fluet GG, Mathai A, Qiu Q, Lewis J, Merians AS (2009). Design of a complex virtual reality simulation to train finger motion for persons with hemiparesis: a proof of concept study. J Neuroeng Rehabil.

[117] Hogan N, Sternad D (2007). On rhythmic and discrete movements: reflections, definitions and implications for motor control. Exp Brain Res.

[118] Huys R, Studenka BE, Rheaume NL, Zelaznik HN, Jirsa VK (2008). Distinct Timing Mechanisms Produce Discrete and Continuous Movements. PLoS Comput Biol.

[119] Schaal S, Sternad D, Osu R, Kawato M (2004). Rhythmic arm movement is not discrete. Nat Neurosci.

[120] Swinnen SP, Jardin K, Verschueren S, Meulenbroek R, Franz L, Dounskaia N, Walter CB (1998). Exploring interlimb constraints during bimanual graphic performance: effects of muscle grouping and direction. Behav Brain Res.

